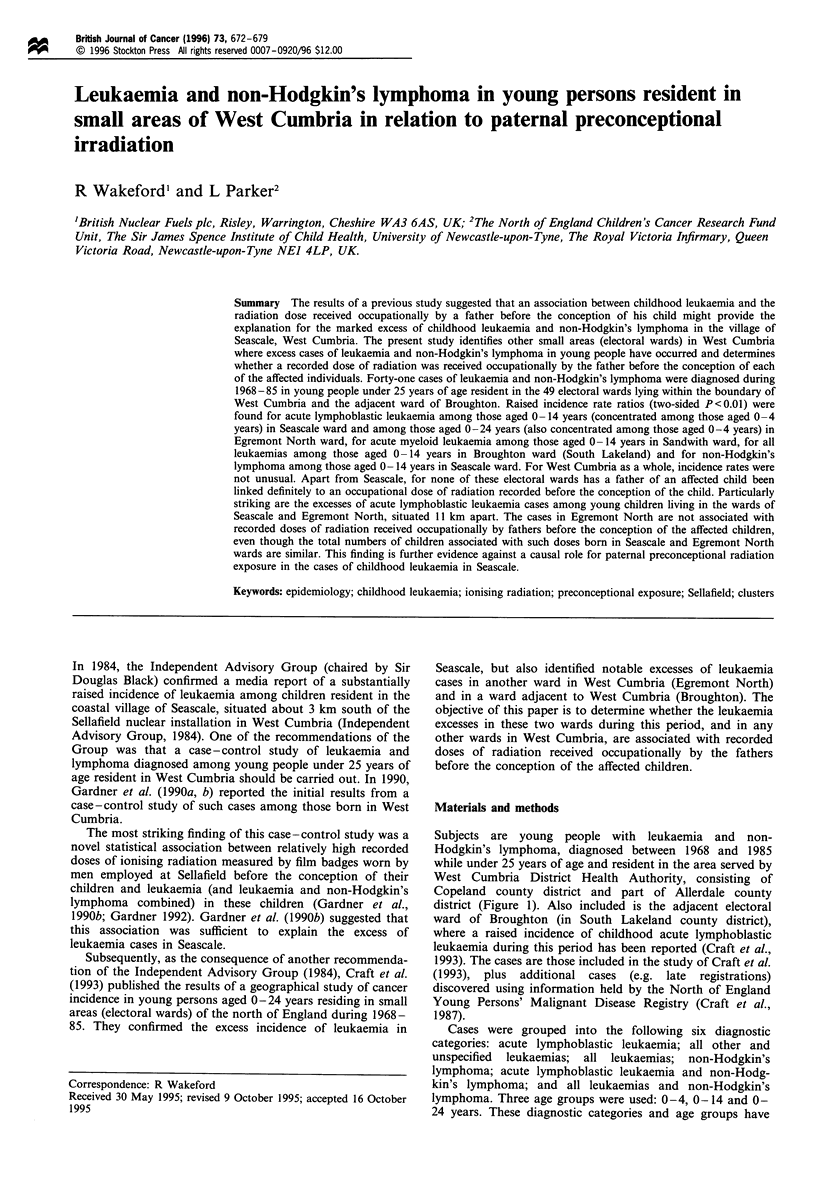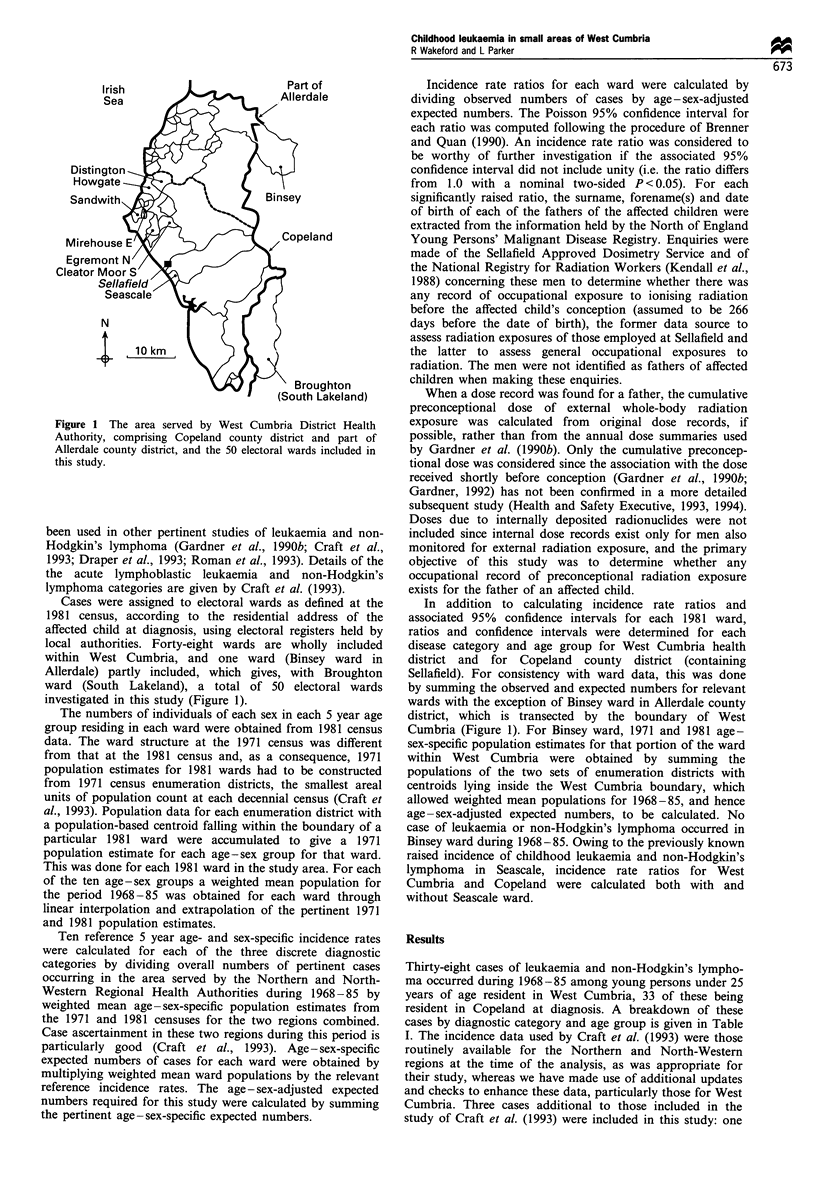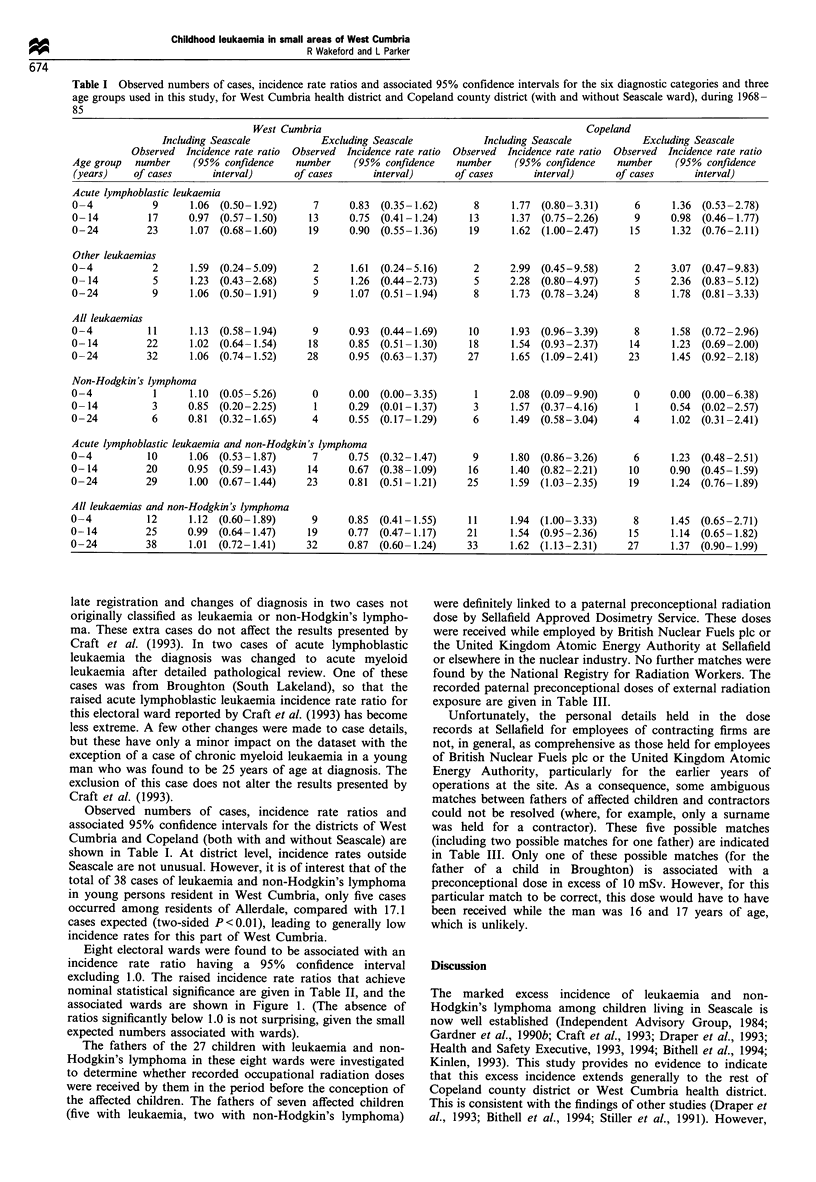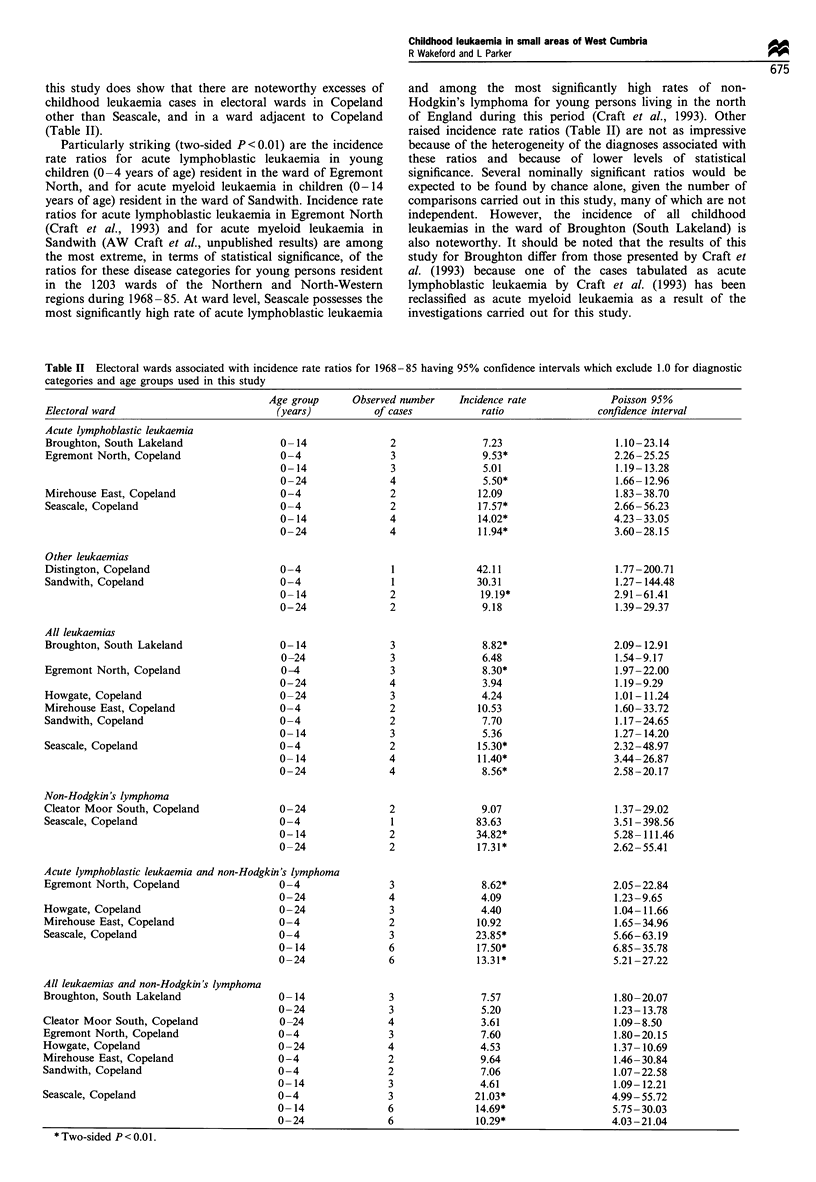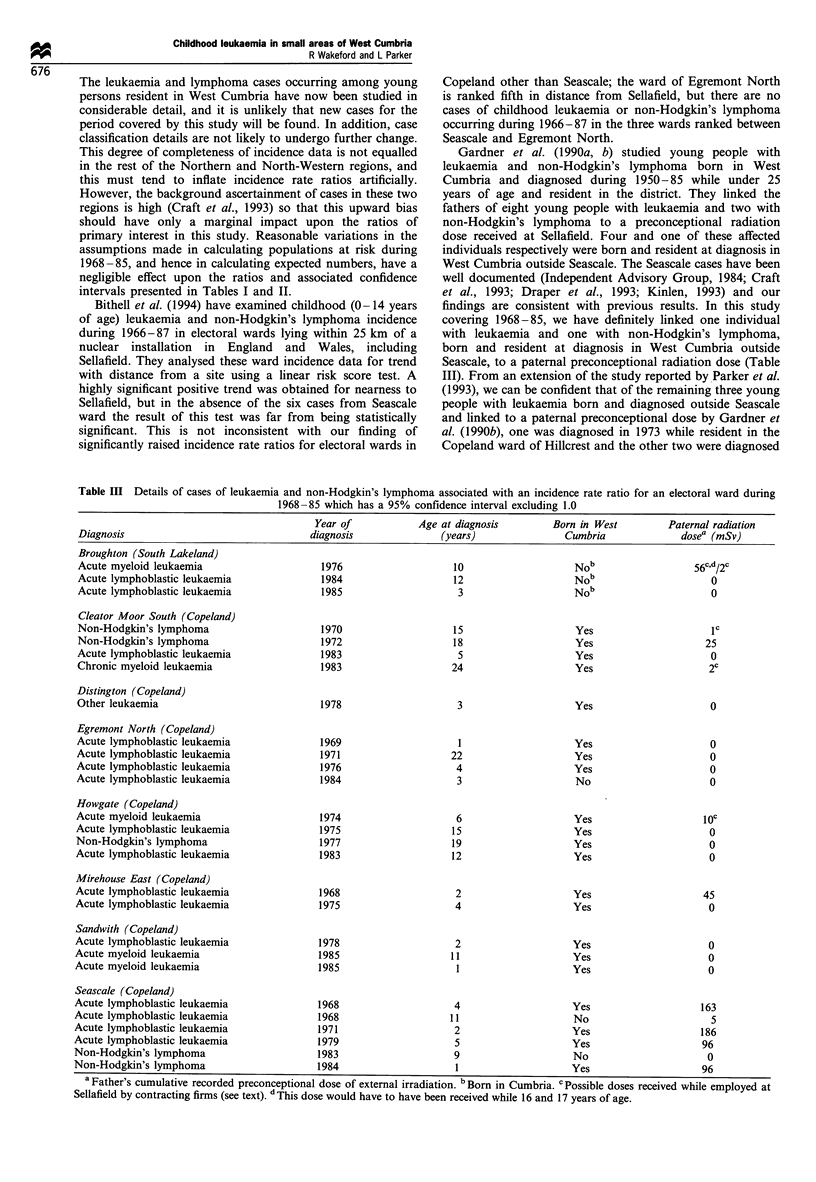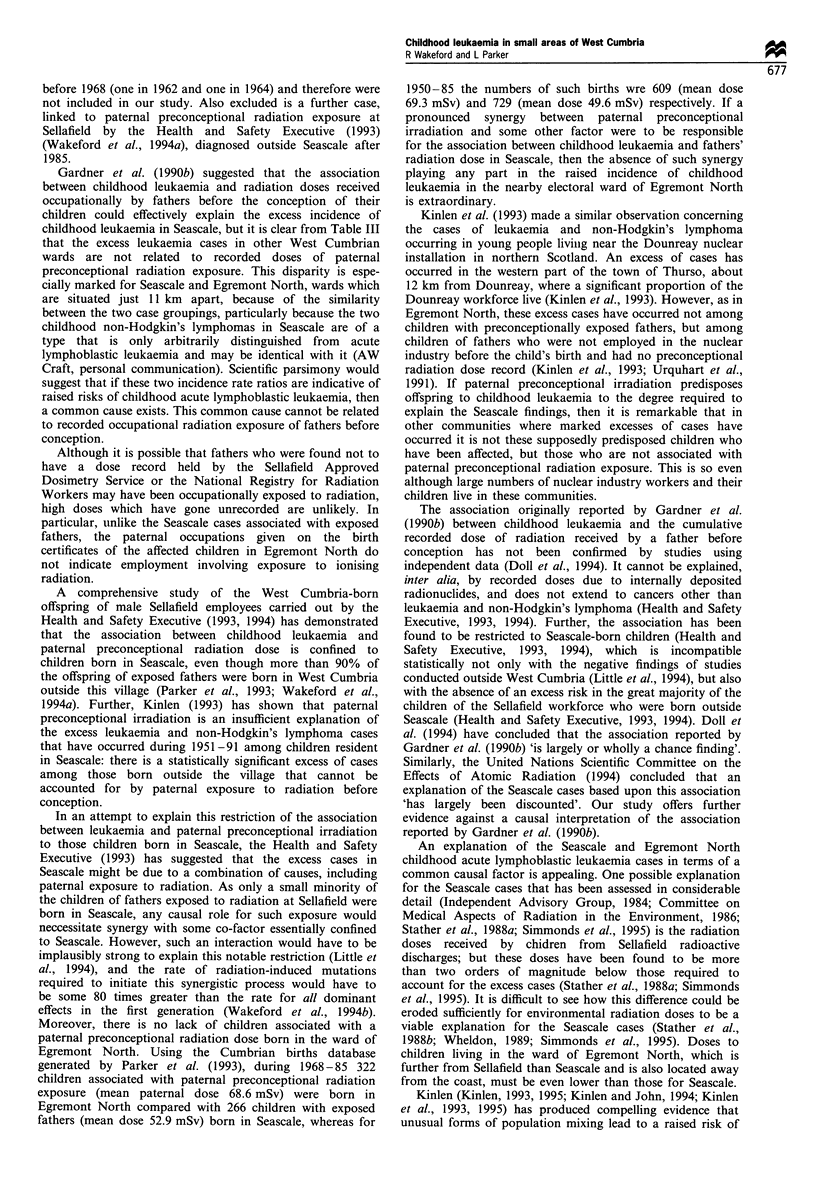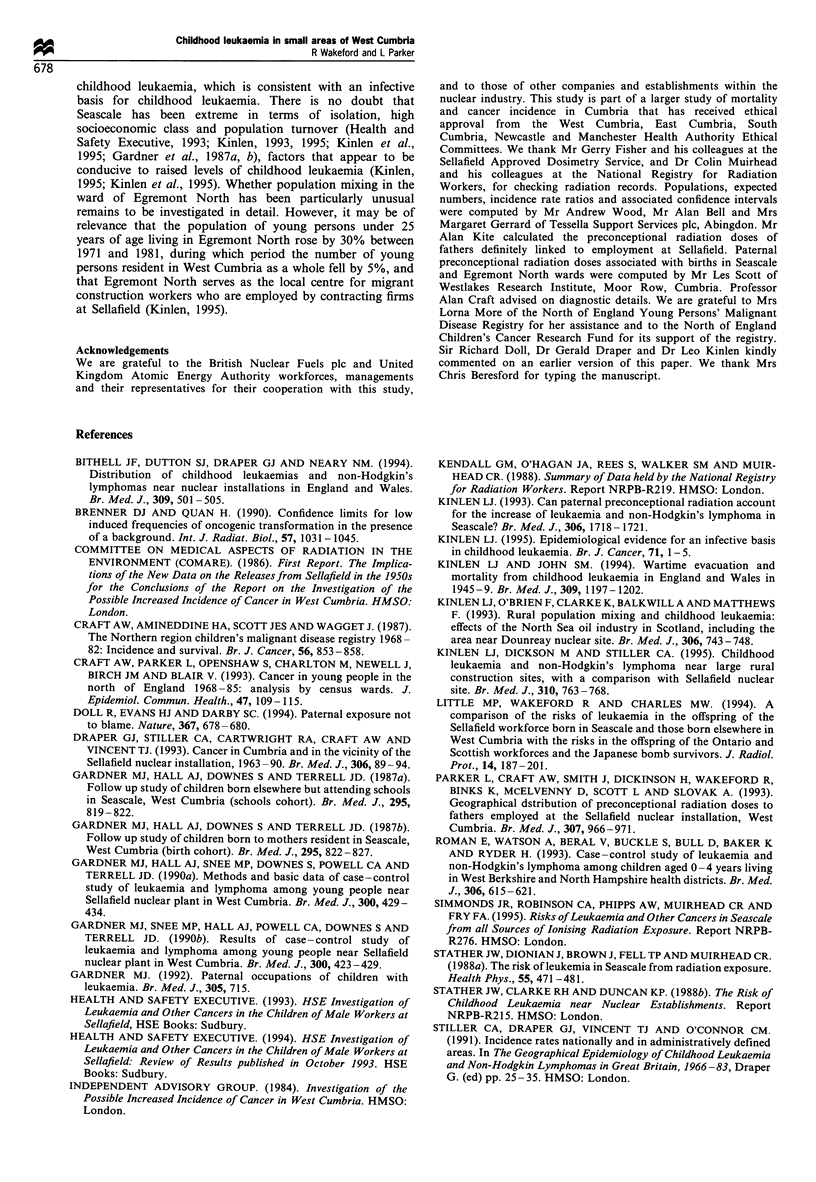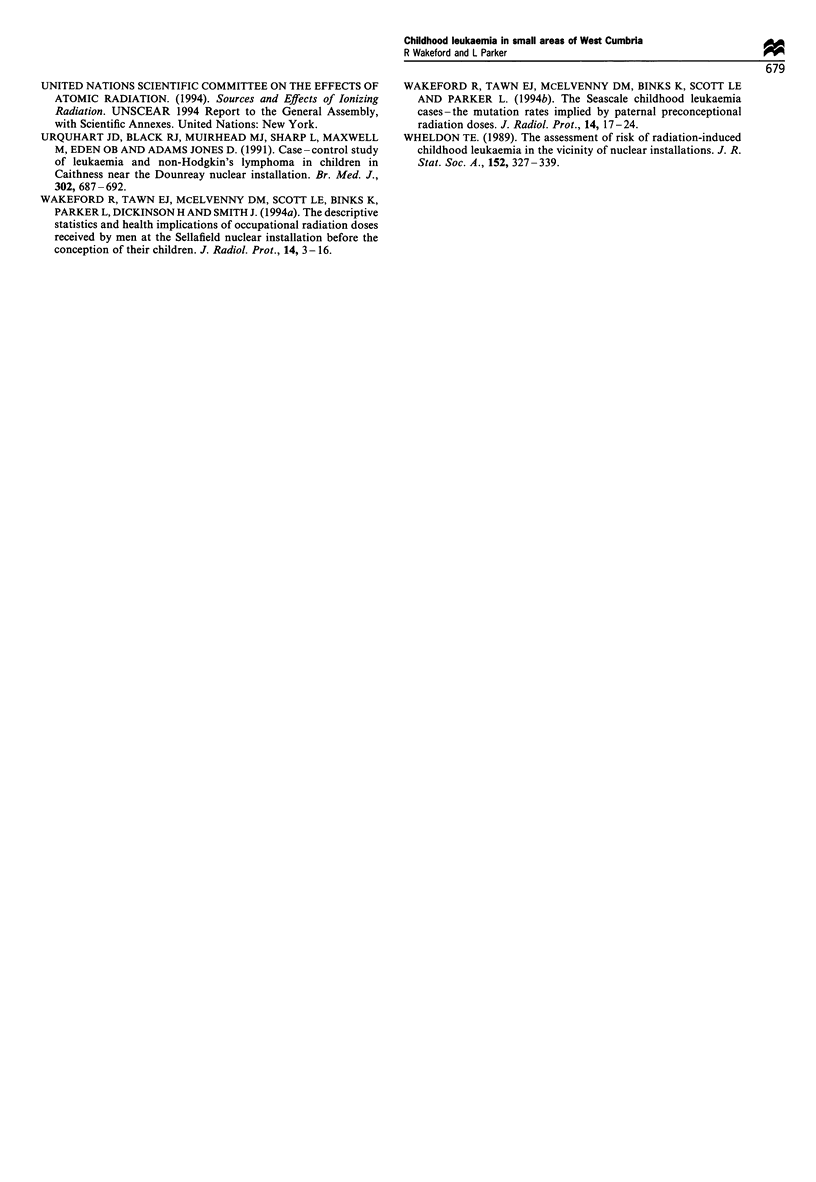# Leukaemia and non-Hodgkin's lymphoma in young persons resident in small areas of West Cumbria in relation to paternal preconceptional irradiation.

**DOI:** 10.1038/bjc.1996.117

**Published:** 1996-03

**Authors:** R. Wakeford, L. Parker

**Affiliations:** British Nuclear Fuels plc, Risley, UK.

## Abstract

The results of a previous study suggested that an association between childhood leukaemia and the radiation dose received occupationally by a father before the conception of his child might provide the explanation for the marked excess of childhood leukaemia and non-Hodgkin's lymphoma in the village of Seascale, West Cumbria. The present study identifies other small areas (electoral wards) in West Cumbria where excess cases of leukaemia and non-Hodgkin's lymphoma in young people have occurred and determines whether a recorded dose of radiation was received occupationally by the father before the conception of each of the affected individuals. Forty-one cases of leukaemia and non-Hodgkin's lymphoma were diagnosed during 1968-85 in young people under 25 years of age resident in the 49 electoral wards lying within the boundary of West Cumbria and the adjacent ward of Broughton. Raised incidence rate ratios (two-sided P<0.01) were found for acute lymphoblastic leukaemia among those aged 0-14 years (concentrated among those aged 0-4 years) in Seascale ward and among those aged 0-24 years (also concentrated among those aged 0-4 years) in Egremont North ward, for acute myeloid leukaemia among those aged 0-14 years in Sandwith ward, for all leukaemias among those aged 0-14 years in Broughton ward (South Lakeland) and for non-Hodgkin's lymphoma among those aged 0-14 years in Seascale ward. For West Cumbria as a whole, incidence rates were not usual. Apart from Seascale, for none of these electoral wards has a father of an affected child been linked definitely to an occupational dose of radiation recorded before the conception of the child. Particularly striking are the excesses of acute lymphoblastic leukaemia cases among young children living in the wards of Seascale and Egremont North, situated 11 km apart. The cases in Egremont North are not associated with recorded doses of radiation received occupationally by fathers before the conception of the affected children, even though the total numbers of children associated with such doses born in Seascale and Egremont North wards are similar. This finding is further evidence against a causal role for paternal preconceptional radiation exposure in the cases of childhood leukaemia in Seascale.


British Journal of Cancer (1996) 73, 672-679
© 1996 Stockton Press All rights reserved 0007-0920/96 $12.00

### Leukaemia and non-Hodgkin's lymphoma in young persons resident in small areas of West Cumbria in relation to paternal preconceptional irradiation

R Wakeford[1] and L Parker[2]

[1]British Nuclear Fuels plc, Risley, Warrington, Cheshire WA3 6AS, UK; [2]The North of England Children's Cancer Research Fund Unit, The Sir James Spence Institute of Child Health, University of Newcastle-upon-Tyne, The Royal Victoria Infirmary, Queen Victoria Road, Newcastle-upon-Tyne NE1 4LP, UK.

**Summary** The results of a previous study suggested that an association between childhood leukaemia and the radiation dose received occupationally by a father before the conception of his child might provide the explanation for the marked excess of childhood leukaemia and non-Hodgkin's lymphoma in the village of Seascale, West Cumbria. The present study identifies other small areas (electoral wards) in West Cumbria where excess cases of leukaemia and non-Hodgkin's lymphoma in young people have occurred and determines whether a recorded dose of radiation was received occupationally by the father before the conception of each of the affected individuals. Forty-one cases of leukaemia and non-Hodgkin's lymphoma were diagnosed during 1968-85 in young people under 25 years of age resident in the 49 electoral wards lying within the boundary of West Cumbria and the adjacent ward of Broughton. Raised incidence rate ratios (two-sided $P<0.01$) were found for acute lymphoblastic leukaemia among those aged 0-14 years (concentrated among those aged 0-4 years) in Seascale ward and among those aged 0-24 years (also concentrated among those aged 0-4 years) in Egremont North ward, for acute myeloid leukaemia among those aged 0-14 years in Sandwith ward, for all leukaemias among those aged 0-14 years in Broughton ward (South Lakeland) and for non-Hodgkin's lymphoma among those aged 0-14 years in Seascale ward. For West Cumbria as a whole, incidence rates were not unusual. Apart from Seascale, for none of these electoral wards has a father of an affected child been linked definitely to an occupational dose of radiation recorded before the conception of the child. Particularly striking are the excesses of acute lymphoblastic leukaemia cases among young children living in the wards of Seascale and Egremont North, situated 11 km apart. The cases in Egremont North are not associated with recorded doses of radiation received occupationally by fathers before the conception of the affected children, even though the total numbers of children associated with such doses born in Seascale and Egremont North wards are similar. This finding is further evidence against a causal role for paternal preconceptional radiation exposure in the cases of childhood leukaemia in Seascale.

**Keywords:** epidemiology; childhood leukaemia; ionising radiation; preconceptional exposure; Sellafield; clusters

In 1984, the Independent Advisory Group (chaired by Sir Douglas Black) confirmed a media report of a substantially raised incidence of leukaemia among children resident in the coastal village of Seascale, situated about 3 km south of the Sellafield nuclear installation in West Cumbria (Independent Advisory Group, 1984). One of the recommendations of the Group was that a case-control study of leukaemia and lymphoma diagnosed among young people under 25 years of age resident in West Cumbria should be carried out. In 1990, Gardner *et al.* (1990*a*, *b*) reported the initial results from a case-control study of such cases among those born in West Cumbria.

The most striking finding of this case-control study was a novel statistical association between relatively high recorded doses of ionising radiation measured by film badges worn by men employed at Sellafield before the conception of their children and leukaemia (and leukaemia and non-Hodgkin's lymphoma combined) in these children (Gardner *et al.*, 1990*b*; Gardner 1992). Gardner *et al.* (1990*b*) suggested that this association was sufficient to explain the excess of leukaemia cases in Seascale.

Subsequently, as the consequence of another recommendation of the Independent Advisory Group (1984), Craft *et al.* (1993) published the results of a geographical study of cancer incidence in young persons aged 0-24 years residing in small areas (electoral wards) of the north of England during 1968-85. They confirmed the excess incidence of leukaemia in Seascale, but also identified notable excesses of leukaemia cases in another ward in West Cumbria (Egremont North) and in a ward adjacent to West Cumbria (Broughton). The objective of this paper is to determine whether the leukaemia excesses in these two wards during this period, and in any other wards in West Cumbria, are associated with recorded doses of radiation received occupationally by the fathers before the conception of the affected children.

### Materials and methods

Subjects are young people with leukaemia and non-Hodgkin's lymphoma, diagnosed between 1968 and 1985 while under 25 years of age and resident in the area served by West Cumbria District Health Authority, consisting of Copeland county district and part of Allerdale county district (Figure 1). Also included is the adjacent electoral ward of Broughton (in South Lakeland county district), where a raised incidence of childhood acute lymphoblastic leukaemia during this period has been reported (Craft *et al.*, 1993). The cases are those included in the study of Craft *et al.* (1993), plus additional cases (e.g. late registrations) discovered using information held by the North of England Young Persons' Malignant Disease Registry (Craft *et al.*, 1987).

Cases were grouped into the following six diagnostic categories: acute lymphoblastic leukaemia; all other and unspecified leukaemias; all leukaemias; non-Hodgkin's lymphoma; acute lymphoblastic leukaemia and non-Hodgkin's lymphoma; and all leukaemias and non-Hodgkin's lymphoma. Three age groups were used: 0-4, 0-14 and 0-24 years. These diagnostic categories and age groups have

Correspondence: R Wakeford
Received 30 May 1995; revised 9 October 1995; accepted 16 October 1995

**Figure 1** The area served by West Cumbria District Health Authority, comprising Copeland county district and part of Allerdale county district, and the 50 electoral wards included in this study.

been used in other pertinent studies of leukaemia and non-Hodgkin's lymphoma (Gardner *et al.*, 1990*b*; Craft *et al.*, 1993; Draper *et al.*, 1993; Roman *et al.*, 1993). Details of the the acute lymphoblastic leukaemia and non-Hodgkin's lymphoma categories are given by Craft *et al.* (1993).

Cases were assigned to electoral wards as defined at the 1981 census, according to the residential address of the affected child at diagnosis, using electoral registers held by local authorities. Forty-eight wards are wholly included within West Cumbria, and one ward (Binsey ward in Allerdale) partly included, which gives, with Broughton ward (South Lakeland), a total of 50 electoral wards investigated in this study (Figure 1).

The numbers of individuals of each sex in each 5 year age group residing in each ward were obtained from 1981 census data. The ward structure at the 1971 census was different from that at the 1981 census and, as a consequence, 1971 population estimates for 1981 wards had to be constructed from 1971 census enumeration districts, the smallest areal units of population count at each decennial census (Craft *et al.*, 1993). Population data for each enumeration district with a population-based centroid falling within the boundary of a particular 1981 ward were accumulated to give a 1971 population estimate for each age–sex group for that ward. This was done for each 1981 ward in the study area. For each of the ten age–sex groups a weighted mean population for the period 1968–85 was obtained for each ward through linear interpolation and extrapolation of the pertinent 1971 and 1981 population estimates.

Ten reference 5 year age- and sex-specific incidence rates were calculated for each of the three discrete diagnostic categories by dividing overall numbers of pertinent cases occurring in the area served by the Northern and North-Western Regional Health Authorities during 1968–85 by weighted mean age–sex-specific population estimates from the 1971 and 1981 censuses for the two regions combined. Case ascertainment in these two regions during this period is particularly good (Craft *et al.*, 1993). Age–sex-specific expected numbers of cases for each ward were obtained by multiplying weighted mean ward populations by the relevant reference incidence rates. The age–sex-adjusted expected numbers required for this study were calculated by summing the pertinent age–sex-specific expected numbers.

Incidence rate ratios for each ward were calculated by dividing observed numbers of cases by age–sex-adjusted expected numbers. The Poisson 95% confidence interval for each ratio was computed following the procedure of Brenner and Quan (1990). An incidence rate ratio was considered to be worthy of further investigation if the associated 95% confidence interval did not include unity (i.e. the ratio differs from 1.0 with a nominal two-sided $P<0.05$). For each significantly raised ratio, the surname, forename(s) and date of birth of each of the fathers of the affected children were extracted from the information held by the North of England Young Persons' Malignant Disease Registry. Enquiries were made of the Sellafield Approved Dosimetry Service and of the National Registry for Radiation Workers (Kendall *et al.*, 1988) concerning these men to determine whether there was any record of occupational exposure to ionising radiation before the affected child's conception (assumed to be 266 days before the date of birth), the former data source to assess radiation exposures of those employed at Sellafield and the latter to assess general occupational exposures to radiation. The men were not identified as fathers of affected children when making these enquiries.

When a dose record was found for a father, the cumulative preconceptional dose of external whole-body radiation exposure was calculated from original dose records, if possible, rather than from the annual dose summaries used by Gardner *et al.* (1990*b*). Only the cumulative preconceptional dose was considered since the association with the dose received shortly before conception (Gardner *et al.*, 1990*b*; Gardner, 1992) has not been confirmed in a more detailed subsequent study (Health and Safety Executive, 1993, 1994). Doses due to internally deposited radionuclides were not included since internal dose records exist only for men also monitored for external radiation exposure, and the primary objective of this study was to determine whether any occupational record of preconceptional radiation exposure exists for the father of an affected child.

In addition to calculating incidence rate ratios and associated 95% confidence intervals for each 1981 ward, ratios and confidence intervals were determined for each disease category and age group for West Cumbria health district and for Copeland county district (containing Sellafield). For consistency with ward data, this was done by summing the observed and expected numbers for relevant wards with the exception of Binsey ward in Allerdale county district, which is transected by the boundary of West Cumbria (Figure 1). For Binsey ward, 1971 and 1981 age–sex-specific population estimates for that portion of the ward within West Cumbria were obtained by summing the populations of the two sets of enumeration districts with centroids lying inside the West Cumbria boundary, which allowed weighted mean populations for 1968–85, and hence age–sex-adjusted expected numbers, to be calculated. No case of leukaemia or non-Hodgkin's lymphoma occurred in Binsey ward during 1968–85. Owing to the previously known raised incidence of childhood leukaemia and non-Hodgkin's lymphoma in Seascale, incidence rate ratios for West Cumbria and Copeland were calculated both with and without Seascale ward.

### Results

Thirty-eight cases of leukaemia and non-Hodgkin's lymphoma occurred during 1968–85 among young persons under 25 years of age resident in West Cumbria, 33 of these being resident in Copeland at diagnosis. A breakdown of these cases by diagnostic category and age group is given in Table I. The incidence data used by Craft *et al.* (1993) were those routinely available for the Northern and North-Western regions at the time of the analysis, as was appropriate for their study, whereas we have made use of additional updates and checks to enhance these data, particularly those for West Cumbria. Three cases additional to those included in the study of Craft *et al.* (1993) were included in this study: one

**Table I** Observed numbers of cases, incidence rate ratios and associated 95% confidence intervals for the six diagnostic categories and three age groups used in this study, for West Cumbria health district and Copeland county district (with and without Seascale ward), during 1968–85

| | *West Cumbria* | | | | *Copeland* | | | |
|---|---|---|---|---|---|---|---|---|
| | *Including Seascale* | | *Excluding Seascale* | | *Including Seascale* | | *Excluding Seascale* | |
| *Age group (years)* | *Observed number of cases* | *Incidence rate ratio (95% confidence interval)* | *Observed number of cases* | *Incidence rate ratio (95% confidence interval)* | *Observed number of cases* | *Incidence rate ratio (95% confidence interval)* | *Observed number of cases* | *Incidence rate ratio (95% confidence interval)* |
| *Acute lymphoblastic leukaemia* | | | | | | | | |
| 0–4 | 9 | 1.06 (0.50–1.92) | 7 | 0.83 (0.35–1.62) | 8 | 1.77 (0.80–3.31) | 6 | 1.36 (0.53–2.78) |
| 0–14 | 17 | 0.97 (0.57–1.50) | 13 | 0.75 (0.41–1.24) | 13 | 1.37 (0.75–2.26) | 9 | 0.98 (0.46–1.77) |
| 0–24 | 23 | 1.07 (0.68–1.60) | 19 | 0.90 (0.55–1.36) | 19 | 1.62 (1.00–2.47) | 15 | 1.32 (0.76–2.11) |
| *Other leukaemias* | | | | | | | | |
| 0–4 | 2 | 1.59 (0.24–5.09) | 2 | 1.61 (0.24–5.16) | 2 | 2.99 (0.45–9.58) | 2 | 3.07 (0.47–9.83) |
| 0–14 | 5 | 1.23 (0.43–2.68) | 5 | 1.26 (0.44–2.73) | 5 | 2.28 (0.80–4.97) | 5 | 2.36 (0.83–5.12) |
| 0–24 | 9 | 1.06 (0.50–1.91) | 9 | 1.07 (0.51–1.94) | 8 | 1.73 (0.78–3.24) | 8 | 1.78 (0.81–3.33) |
| *All leukaemias* | | | | | | | | |
| 0–4 | 11 | 1.13 (0.58–1.94) | 9 | 0.93 (0.44–1.69) | 10 | 1.93 (0.96–3.39) | 8 | 1.58 (0.72–2.96) |
| 0–14 | 22 | 1.02 (0.64–1.54) | 18 | 0.85 (0.51–1.30) | 18 | 1.54 (0.93–2.37) | 14 | 1.23 (0.69–2.00) |
| 0–24 | 32 | 1.06 (0.74–1.52) | 28 | 0.95 (0.63–1.37) | 27 | 1.65 (1.09–2.41) | 23 | 1.45 (0.92–2.18) |
| *Non-Hodgkin's lymphoma* | | | | | | | | |
| 0–4 | 1 | 1.10 (0.05–5.26) | 0 | 0.00 (0.00–3.35) | 1 | 2.08 (0.09–9.90) | 0 | 0.00 (0.00–6.38) |
| 0–14 | 3 | 0.85 (0.20–2.25) | 1 | 0.29 (0.01–1.37) | 3 | 1.57 (0.37–4.16) | 1 | 0.54 (0.02–2.57) |
| 0–24 | 6 | 0.81 (0.32–1.65) | 4 | 0.55 (0.17–1.29) | 6 | 1.49 (0.58–3.04) | 4 | 1.02 (0.31–2.41) |
| *Acute lymphoblastic leukaemia and non-Hodgkin's lymphoma* | | | | | | | | |
| 0–4 | 10 | 1.06 (0.53–1.87) | 7 | 0.75 (0.32–1.47) | 9 | 1.80 (0.86–3.26) | 6 | 1.23 (0.48–2.51) |
| 0–14 | 20 | 0.95 (0.59–1.43) | 14 | 0.67 (0.38–1.09) | 16 | 1.40 (0.82–2.21) | 10 | 0.90 (0.45–1.59) |
| 0–24 | 29 | 1.00 (0.67–1.44) | 23 | 0.81 (0.51–1.21) | 25 | 1.59 (1.03–2.35) | 19 | 1.24 (0.76–1.89) |
| *All leukaemias and non-Hodgkin's lymphoma* | | | | | | | | |
| 0–4 | 12 | 1.12 (0.60–1.89) | 9 | 0.85 (0.41–1.55) | 11 | 1.94 (1.00–3.33) | 8 | 1.45 (0.65–2.71) |
| 0–14 | 25 | 0.99 (0.64–1.47) | 19 | 0.77 (0.47–1.17) | 21 | 1.54 (0.95–2.36) | 15 | 1.14 (0.65–1.82) |
| 0–24 | 38 | 1.01 (0.72–1.41) | 32 | 0.87 (0.60–1.24) | 33 | 1.62 (1.13–2.31) | 27 | 1.37 (0.90–1.99) |

late registration and changes of diagnosis in two cases not originally classified as leukaemia or non-Hodgkin's lymphoma. These extra cases do not affect the results presented by Craft *et al.* (1993). In two cases of acute lymphoblastic leukaemia the diagnosis was changed to acute myeloid leukaemia after detailed pathological review. One of these cases was from Broughton (South Lakeland), so that the raised acute lymphoblastic leukaemia incidence rate ratio for this electoral ward reported by Craft *et al.* (1993) has become less extreme. A few other changes were made to case details, but these have only a minor impact on the dataset with the exception of a case of chronic myeloid leukaemia in a young man who was found to be 25 years of age at diagnosis. The exclusion of this case does not alter the results presented by Craft *et al.* (1993).

Observed numbers of cases, incidence rate ratios and associated 95% confidence intervals for the districts of West Cumbria and Copeland (both with and without Seascale) are shown in Table I. At district level, incidence rates outside Seascale are not unusual. However, it is of interest that of the total of 38 cases of leukaemia and non-Hodgkin's lymphoma in young persons resident in West Cumbria, only five cases occurred among residents of Allerdale, compared with 17.1 cases expected (two-sided $P < 0.01$), leading to generally low incidence rates for this part of West Cumbria.

Eight electoral wards were found to be associated with an incidence rate ratio having a 95% confidence interval excluding 1.0. The raised incidence rate ratios that achieve nominal statistical significance are given in Table II, and the associated wards are shown in Figure 1. (The absence of ratios significantly below 1.0 is not surprising, given the small expected numbers associated with wards).

The fathers of the 27 children with leukaemia and non-Hodgkin's lymphoma in these eight wards were investigated to determine whether recorded occupational radiation doses were received by them in the period before the conception of the affected children. The fathers of seven affected children (five with leukaemia, two with non-Hodgkin's lymphoma) were definitely linked to a paternal preconceptional radiation dose by Sellafield Approved Dosimetry Service. These doses were received while employed by British Nuclear Fuels plc or the United Kingdom Atomic Energy Authority at Sellafield or elsewhere in the nuclear industry. No further matches were found by the National Registry for Radiation Workers. The recorded paternal preconceptional doses of external radiation exposure are given in Table III.

Unfortunately, the personal details held in the dose records at Sellafield for employees of contracting firms are not, in general, as comprehensive as those held for employees of British Nuclear Fuels plc or the United Kingdom Atomic Energy Authority, particularly for the earlier years of operations at the site. As a consequence, some ambiguous matches between fathers of affected children and contractors could not be resolved (where, for example, only a surname was held for a contractor). These five possible matches (including two possible matches for one father) are indicated in Table III. Only one of these possible matches (for the father of a child in Broughton) is associated with a preconceptional dose in excess of 10 mSv. However, for this particular match to be correct, this dose would have to have been received while the man was 16 and 17 years of age, which is unlikely.

### Discussion

The marked excess incidence of leukaemia and non-Hodgkin's lymphoma among children living in Seascale is now well established (Independent Advisory Group, 1984; Gardner *et al.*, 1990*b*; Craft *et al.*, 1993; Draper *et al.*, 1993; Health and Safety Executive, 1993, 1994; Bithell *et al.*, 1994; Kinlen, 1993). This study provides no evidence to indicate that this excess incidence extends generally to the rest of Copeland county district or West Cumbria health district. This is consistent with the findings of other studies (Draper *et al.*, 1993; Bithell *et al.*, 1994; Stiller *et al.*, 1991). However, this study does show that there are noteworthy excesses of childhood leukaemia cases in electoral wards in Copeland other than Seascale, and in a ward adjacent to Copeland (Table II).

Particularly striking (two-sided $P<0.01$) are the incidence rate ratios for acute lymphoblastic leukaemia in young children (0–4 years of age) resident in the ward of Egremont North, and for acute myeloid leukaemia in children (0–14 years of age) resident in the ward of Sandwith. Incidence rate ratios for acute lymphoblastic leukaemia in Egremont North (Craft *et al.*, 1993) and for acute myeloid leukaemia in Sandwith (AW Craft *et al.*, unpublished results) are among the most extreme, in terms of statistical significance, of the ratios for these disease categories for young persons resident in the 1203 wards of the Northern and North-Western regions during 1968–85. At ward level, Seascale possesses the most significantly high rate of acute lymphoblastic leukaemia and among the most significantly high rates of non-Hodgkin's lymphoma for young persons living in the north of England during this period (Craft *et al.*, 1993). Other raised incidence rate ratios (Table II) are not as impressive because of the heterogeneity of the diagnoses associated with these ratios and because of lower levels of statistical significance. Several nominally significant ratios would be expected to be found by chance alone, given the number of comparisons carried out in this study, many of which are not independent. However, the incidence of all childhood leukaemias in the ward of Broughton (South Lakeland) is also noteworthy. It should be noted that the results of this study for Broughton differ from those presented by Craft *et al.* (1993) because one of the cases tabulated as acute lymphoblastic leukaemia by Craft *et al.* (1993) has been reclassified as acute myeloid leukaemia as a result of the investigations carried out for this study.

**Table II** Electoral wards associated with incidence rate ratios for 1968–85 having 95% confidence intervals which exclude 1.0 for diagnostic categories and age groups used in this study

| *Electoral ward* | *Age group (years)* | *Observed number of cases* | *Incidence rate ratio* | *Poisson 95% confidence interval* |
|---|---|---|---|---|
| *Acute lymphoblastic leukaemia* | | | | |
| Broughton, South Lakeland | 0–14 | 2 | 7.23 | 1.10–23.14 |
| Egremont North, Copeland | 0–4 | 3 | 9.53* | 2.26–25.25 |
| | 0–14 | 3 | 5.01 | 1.19–13.28 |
| | 0–24 | 4 | 5.50* | 1.66–12.96 |
| Mirehouse East, Copeland | 0–4 | 2 | 12.09 | 1.83–38.70 |
| Seascale, Copeland | 0–4 | 2 | 17.57* | 2.66–56.23 |
| | 0–14 | 4 | 14.02* | 4.23–33.05 |
| | 0–24 | 4 | 11.94* | 3.60–28.15 |
| *Other leukaemias* | | | | |
| Distington, Copeland | 0–4 | 1 | 42.11 | 1.77–200.71 |
| Sandwith, Copeland | 0–4 | 1 | 30.31 | 1.27–144.48 |
| | 0–14 | 2 | 19.19* | 2.91–61.41 |
| | 0–24 | 2 | 9.18 | 1.39–29.37 |
| *All leukaemias* | | | | |
| Broughton, South Lakeland | 0–14 | 3 | 8.82* | 2.09–12.91 |
| | 0–24 | 3 | 6.48 | 1.54–9.17 |
| Egremont North, Copeland | 0–4 | 3 | 8.30* | 1.97–22.00 |
| | 0–24 | 4 | 3.94 | 1.19–9.29 |
| Howgate, Copeland | 0–24 | 3 | 4.24 | 1.01–11.24 |
| Mirehouse East, Copeland | 0–4 | 2 | 10.53 | 1.60–33.72 |
| Sandwith, Copeland | 0–4 | 2 | 7.70 | 1.17–24.65 |
| | 0–14 | 3 | 5.36 | 1.27–14.20 |
| Seascale, Copeland | 0–4 | 2 | 15.30* | 2.32–48.97 |
| | 0–14 | 4 | 11.40* | 3.44–26.87 |
| | 0–24 | 4 | 8.56* | 2.58–20.17 |
| *Non-Hodgkin's lymphoma* | | | | |
| Cleator Moor South, Copeland | 0–24 | 2 | 9.07 | 1.37–29.02 |
| Seascale, Copeland | 0–4 | 1 | 83.63 | 3.51–398.56 |
| | 0–14 | 2 | 34.82* | 5.28–111.46 |
| | 0–24 | 2 | 17.31* | 2.62–55.41 |
| *Acute lymphoblastic leukaemia and non-Hodgkin's lymphoma* | | | | |
| Egremont North, Copeland | 0–4 | 3 | 8.62* | 2.05–22.84 |
| | 0–24 | 4 | 4.09 | 1.23–9.65 |
| Howgate, Copeland | 0–24 | 3 | 4.40 | 1.04–11.66 |
| Mirehouse East, Copeland | 0–4 | 2 | 10.92 | 1.65–34.96 |
| Seascale, Copeland | 0–4 | 3 | 23.85* | 5.66–63.19 |
| | 0–14 | 6 | 17.50* | 6.85–35.78 |
| | 0–24 | 6 | 13.31* | 5.21–27.22 |
| *All leukaemias and non-Hodgkin's lymphoma* | | | | |
| Broughton, South Lakeland | 0–14 | 3 | 7.57 | 1.80–20.07 |
| | 0–24 | 3 | 5.20 | 1.23–13.78 |
| Cleator Moor South, Copeland | 0–24 | 4 | 3.61 | 1.09–8.50 |
| Egremont North, Copeland | 0–4 | 3 | 7.60 | 1.80–20.15 |
| Howgate, Copeland | 0–24 | 4 | 4.53 | 1.37–10.69 |
| Mirehouse East, Copeland | 0–4 | 2 | 9.64 | 1.46–30.84 |
| Sandwith, Copeland | 0–4 | 2 | 7.06 | 1.07–22.58 |
| | 0–14 | 3 | 4.61 | 1.09–12.21 |
| Seascale, Copeland | 0–4 | 3 | 21.03* | 4.99–55.72 |
| | 0–14 | 6 | 14.69* | 5.75–30.03 |
| | 0–24 | 6 | 10.29* | 4.03–21.04 |

*Two-sided $P<0.01$.

The leukaemia and lymphoma cases occurring among young persons resident in West Cumbria have now been studied in considerable detail, and it is unlikely that new cases for the period covered by this study will be found. In addition, case classification details are not likely to undergo further change. This degree of completeness of incidence data is not equalled in the rest of the Northern and North-Western regions, and this must tend to inflate incidence rate ratios artificially. However, the background ascertainment of cases in these two regions is high (Craft *et al.*, 1993) so that this upward bias should have only a marginal impact upon the ratios of primary interest in this study. Reasonable variations in the assumptions made in calculating populations at risk during 1968–85, and hence in calculating expected numbers, have a negligible effect upon the ratios and associated confidence intervals presented in Tables I and II.

Bithell *et al.* (1994) have examined childhood (0–14 years of age) leukaemia and non-Hodgkin's lymphoma incidence during 1966–87 in electoral wards lying within 25 km of a nuclear installation in England and Wales, including Sellafield. They analysed these ward incidence data for trend with distance from a site using a linear risk score test. A highly significant positive trend was obtained for nearness to Sellafield, but in the absence of the six cases from Seascale ward the result of this test was far from being statistically significant. This is not inconsistent with our finding of significantly raised incidence rate ratios for electoral wards in Copeland other than Seascale; the ward of Egremont North is ranked fifth in distance from Sellafield, but there are no cases of childhood leukaemia or non-Hodgkin's lymphoma occurring during 1966–87 in the three wards ranked between Seascale and Egremont North.

Gardner *et al.* (1990*a*, *b*) studied young people with leukaemia and non-Hodgkin's lymphoma born in West Cumbria and diagnosed during 1950–85 while under 25 years of age and resident in the district. They linked the fathers of eight young people with leukaemia and two with non-Hodgkin's lymphoma to a preconceptional radiation dose received at Sellafield. Four and one of these affected individuals respectively were born and resident at diagnosis in West Cumbria outside Seascale. The Seascale cases have been well documented (Independent Advisory Group, 1984; Craft *et al.*, 1993; Draper *et al.*, 1993; Kinlen, 1993) and our findings are consistent with previous results. In this study covering 1968–85, we have definitely linked one individual with leukaemia and one with non-Hodgkin's lymphoma, born and resident at diagnosis in West Cumbria outside Seascale, to a paternal preconceptional radiation dose (Table III). From an extension of the study reported by Parker *et al.* (1993), we can be confident that of the remaining three young people with leukaemia born and diagnosed outside Seascale and linked to a paternal preconceptional dose by Gardner *et al.* (1990*b*), one was diagnosed in 1973 while resident in the Copeland ward of Hillcrest and the other two were diagnosed

**Table III** Details of cases of leukaemia and non-Hodgkin's lymphoma associated with an incidence rate ratio for an electoral ward during 1968–85 which has a 95% confidence interval excluding 1.0

| *Diagnosis* | *Year of diagnosis* | *Age at diagnosis (years)* | *Born in West Cumbria* | *Paternal radiation dose[a] (mSv)* |
|---|---|---|---|---|
| *Broughton (South Lakeland)* | | | | |
| Acute myeloid leukaemia | 1976 | 10 | No[b] | 56[c,d]/2[c] |
| Acute lymphoblastic leukaemia | 1984 | 12 | No[b] | 0 |
| Acute lymphoblastic leukaemia | 1985 | 3 | No[b] | 0 |
| *Cleator Moor South (Copeland)* | | | | |
| Non-Hodgkin's lymphoma | 1970 | 15 | Yes | 1[c] |
| Non-Hodgkin's lymphoma | 1972 | 18 | Yes | 25 |
| Acute lymphoblastic leukaemia | 1983 | 5 | Yes | 0 |
| Chronic myeloid leukaemia | 1983 | 24 | Yes | 2[c] |
| *Distington (Copeland)* | | | | |
| Other leukaemia | 1978 | 3 | Yes | 0 |
| *Egremont North (Copeland)* | | | | |
| Acute lymphoblastic leukaemia | 1969 | 1 | Yes | 0 |
| Acute lymphoblastic leukaemia | 1971 | 22 | Yes | 0 |
| Acute lymphoblastic leukaemia | 1976 | 4 | Yes | 0 |
| Acute lymphoblastic leukaemia | 1984 | 3 | No | 0 |
| *Howgate (Copeland)* | | | | |
| Acute myeloid leukaemia | 1974 | 6 | Yes | 10[c] |
| Acute lymphoblastic leukaemia | 1975 | 15 | Yes | 0 |
| Non-Hodgkin's lymphoma | 1977 | 19 | Yes | 0 |
| Acute lymphoblastic leukaemia | 1983 | 12 | Yes | 0 |
| *Mirehouse East (Copeland)* | | | | |
| Acute lymphoblastic leukaemia | 1968 | 2 | Yes | 45 |
| Acute lymphoblastic leukaemia | 1975 | 4 | Yes | 0 |
| *Sandwith (Copeland)* | | | | |
| Acute lymphoblastic leukaemia | 1978 | 2 | Yes | 0 |
| Acute myeloid leukaemia | 1985 | 11 | Yes | 0 |
| Acute myeloid leukaemia | 1985 | 1 | Yes | 0 |
| *Seascale (Copeland)* | | | | |
| Acute lymphoblastic leukaemia | 1968 | 4 | Yes | 163 |
| Acute lymphoblastic leukaemia | 1968 | 11 | No | 5 |
| Acute lymphoblastic leukaemia | 1971 | 2 | Yes | 186 |
| Acute lymphoblastic leukaemia | 1979 | 5 | Yes | 96 |
| Non-Hodgkin's lymphoma | 1983 | 9 | No | 0 |
| Non-Hodgkin's lymphoma | 1984 | 1 | Yes | 96 |

[a] Father's cumulative recorded preconceptional dose of external irradiation. [b] Born in Cumbria. [c] Possible doses received while employed at Sellafield by contracting firms (see text). [d] This dose would have to have been received while 16 and 17 years of age.

before 1968 (one in 1962 and one in 1964) and therefore were not included in our study. Also excluded is a further case, linked to paternal preconceptional radiation exposure at Sellafield by the Health and Safety Executive (1993) (Wakeford *et al.*, 1994*a*), diagnosed outside Seascale after 1985.

Gardner *et al.* (1990*b*) suggested that the association between childhood leukaemia and radiation doses received occupationally by fathers before the conception of their children could effectively explain the excess incidence of childhood leukaemia in Seascale, but it is clear from Table III that the excess leukaemia cases in other West Cumbrian wards are not related to recorded doses of paternal preconceptional radiation exposure. This disparity is especially marked for Seascale and Egremont North, wards which are situated just 11 km apart, because of the similarity between the two case groupings, particularly because the two childhood non-Hodgkin's lymphomas in Seascale are of a type that is only arbitrarily distinguished from acute lymphoblastic leukaemia and may be identical with it (AW Craft, personal communication). Scientific parsimony would suggest that if these two incidence rate ratios are indicative of raised risks of childhood acute lymphoblastic leukaemia, then a common cause exists. This common cause cannot be related to recorded occupational radiation exposure of fathers before conception.

Although it is possible that fathers who were found not to have a dose record held by the Sellafield Approved Dosimetry Service or the National Registry for Radiation Workers may have been occupationally exposed to radiation, high doses which have gone unrecorded are unlikely. In particular, unlike the Seascale cases associated with exposed fathers, the paternal occupations given on the birth certificates of the affected children in Egremont North do not indicate employment involving exposure to ionising radiation.

A comprehensive study of the West Cumbria-born offspring of male Sellafield employees carried out by the Health and Safety Executive (1993, 1994) has demonstrated that the association between childhood leukaemia and paternal preconceptional radiation dose is confined to children born in Seascale, even though more than 90% of the offspring of exposed fathers were born in West Cumbria outside this village (Parker *et al.*, 1993; Wakeford *et al.*, 1994*a*). Further, Kinlen (1993) has shown that paternal preconceptional irradiation is an insufficient explanation of the excess leukaemia and non-Hodgkin's lymphoma cases that have occurred during 1951–91 among children resident in Seascale: there is a statistically significant excess of cases among those born outside the village that cannot be accounted for by paternal exposure to radiation before conception.

In an attempt to explain this restriction of the association between leukaemia and paternal preconceptional irradiation to those children born in Seascale, the Health and Safety Executive (1993) has suggested that the excess cases in Seascale might be due to a combination of causes, including paternal exposure to radiation. As only a small minority of the children of fathers exposed to radiation at Sellafield were born in Seascale, any causal role for such exposure would neccessitate synergy with some co-factor essentially confined to Seascale. However, such an interaction would have to be implausibly strong to explain this notable restriction (Little *et al.*, 1994), and the rate of radiation-induced mutations required to initiate this synergistic process would have to be some 80 times greater than the rate for *all* dominant effects in the first generation (Wakeford *et al.*, 1994*b*). Moreover, there is no lack of children associated with a paternal preconceptional radiation dose born in the ward of Egremont North. Using the Cumbrian births database generated by Parker *et al.* (1993), during 1968–85 322 children associated with paternal preconceptional radiation exposure (mean paternal dose 68.6 mSv) were born in Egremont North compared with 266 children with exposed fathers (mean dose 52.9 mSv) born in Seascale, whereas for 1950–85 the numbers of such births wre 609 (mean dose 69.3 mSv) and 729 (mean dose 49.6 mSv) respectively. If a pronounced synergy between paternal preconceptional irradiation and some other factor were to be responsible for the association between childhood leukaemia and fathers' radiation dose in Seascale, then the absence of such synergy playing any part in the raised incidence of childhood leukaemia in the nearby electoral ward of Egremont North is extraordinary.

Kinlen *et al.* (1993) made a similar observation concerning the cases of leukaemia and non-Hodgkin's lymphoma occurring in young people living near the Dounreay nuclear installation in northern Scotland. An excess of cases has occurred in the western part of the town of Thurso, about 12 km from Dounreay, where a significant proportion of the Dounreay workforce live (Kinlen *et al.*, 1993). However, as in Egremont North, these excess cases have occurred not among children with preconceptionally exposed fathers, but among children of fathers who were not employed in the nuclear industry before the child's birth and had no preconceptional radiation dose record (Kinlen *et al.*, 1993; Urquhart *et al.*, 1991). If paternal preconceptional irradiation predisposes offspring to childhood leukaemia to the degree required to explain the Seascale findings, then it is remarkable that in other communities where marked excesses of cases have occurred it is not these supposedly predisposed children who have been affected, but those who are not associated with paternal preconceptional radiation exposure. This is so even although large numbers of nuclear industry workers and their children live in these communities.

The association originally reported by Gardner *et al.* (1990*b*) between childhood leukaemia and the cumulative recorded dose of radiation received by a father before conception has not been confirmed by studies using independent data (Doll *et al.*, 1994). It cannot be explained, *inter alia*, by recorded doses due to internally deposited radionuclides, and does not extend to cancers other than leukaemia and non-Hodgkin's lymphoma (Health and Safety Executive, 1993, 1994). Further, the association has been found to be restricted to Seascale-born children (Health and Safety Executive, 1993, 1994), which is incompatible statistically not only with the negative findings of studies conducted outside West Cumbria (Little *et al.*, 1994), but also with the absence of an excess risk in the great majority of the children of the Sellafield workforce who were born outside Seascale (Health and Safety Executive, 1993, 1994). Doll *et al.* (1994) have concluded that the association reported by Gardner *et al.* (1990*b*) 'is largely or wholly a chance finding'. Similarly, the United Nations Scientific Committee on the Effects of Atomic Radiation (1994) concluded that an explanation of the Seascale cases based upon this association 'has largely been discounted'. Our study offers further evidence against a causal interpretation of the association reported by Gardner *et al.* (1990*b*).

An explanation of the Seascale and Egremont North childhood acute lymphoblastic leukaemia cases in terms of a common causal factor is appealing. One possible explanation for the Seascale cases that has been assessed in considerable detail (Independent Advisory Group, 1984; Committee on Medical Aspects of Radiation in the Environment, 1986; Stather *et al.*, 1988*a*; Simmonds *et al.*, 1995) is the radiation doses received by children from Sellafield radioactive discharges; but these doses have been found to be more than two orders of magnitude below those required to account for the excess cases (Stather *et al.*, 1988*a*; Simmonds *et al.*, 1995). It is difficult to see how this difference could be eroded sufficiently for environmental radiation doses to be a viable explanation for the Seascale cases (Stather *et al.*, 1988*b*; Wheldon, 1989; Simmonds *et al.*, 1995). Doses to children living in the ward of Egremont North, which is further from Sellafield than Seascale and is also located away from the coast, must be even lower than those for Seascale.

Kinlen (Kinlen, 1993, 1995; Kinlen and John, 1994; Kinlen *et al.*, 1993, 1995) has produced compelling evidence that unusual forms of population mixing lead to a raised risk of childhood leukaemia, which is consistent with an infective basis for childhood leukaemia. There is no doubt that Seascale has been extreme in terms of isolation, high socioeconomic class and population turnover (Health and Safety Executive, 1993; Kinlen, 1993, 1995; Kinlen *et al.*, 1995; Gardner *et al.*, 1987*a*, *b*), factors that appear to be conducive to raised levels of childhood leukaemia (Kinlen, 1995; Kinlen *et al.*, 1995). Whether population mixing in the ward of Egremont North has been particularly unusual remains to be investigated in detail. However, it may be of relevance that the population of young persons under 25 years of age living in Egremont North rose by 30% between 1971 and 1981, during which period the number of young persons resident in West Cumbria as a whole fell by 5%, and that Egremont North serves as the local centre for migrant construction workers who are employed by contracting firms at Sellafield (Kinlen, 1995).

**Acknowledgements**
We are grateful to the British Nuclear Fuels plc and United Kingdom Atomic Energy Authority workforces, managements and their representatives for their cooperation with this study, and to those of other companies and establishments within the nuclear industry. This study is part of a larger study of mortality and cancer incidence in Cumbria that has received ethical approval from the West Cumbria, East Cumbria, South Cumbria, Newcastle and Manchester Health Authority Ethical Committees. We thank Mr Gerry Fisher and his colleagues at the Sellafield Approved Dosimetry Service, and Dr Colin Muirhead and his colleagues at the National Registry for Radiation Workers, for checking radiation records. Populations, expected numbers, incidence rate ratios and associated confidence intervals were computed by Mr Andrew Wood, Mr Alan Bell and Mrs Margaret Gerrard of Tessella Support Services plc, Abingdon. Mr Alan Kite calculated the preconceptional radiation doses of fathers definitely linked to employment at Sellafield. Paternal preconceptional radiation doses associated with births in Seascale and Egremont North wards were computed by Mr Les Scott of Westlakes Research Institute, Moor Row, Cumbria. Professor Alan Craft advised on diagnostic details. We are grateful to Mrs Lorna More of the North of England Young Persons' Malignant Disease Registry for her assistance and to the North of England Children's Cancer Research Fund for its support of the registry. Sir Richard Doll, Dr Gerald Draper and Dr Leo Kinlen kindly commented on an earlier version of this paper. We thank Mrs Chris Beresford for typing the manuscript.

### References

BITHELL JF, DUTTON SJ, DRAPER GJ AND NEARY NM. (1994). Distribution of childhood leukaemias and non-Hodgkin's lymphomas near nuclear installations in England and Wales. *Br. Med. J.*, **309**, 501–505.

BRENNER DJ AND QUAN H. (1990). Confidence limits for low induced frequencies of oncogenic transformation in the presence of a background. *Int. J. Radiat. Biol.*, **57**, 1031–1045.

COMMITTEE ON MEDICAL ASPECTS OF RADIATION IN THE ENVIRONMENT (COMARE). (1986). *First Report. The Implications of the New Data on the Releases from Sellafield in the 1950s for the Conclusions of the Report on the Investigation of the Possible Increased Incidence of Cancer in West Cumbria. HMSO: London.*

CRAFT AW, AMINEDDINE HA, SCOTT JES AND WAGGET J. (1987). The Northern region children's malignant disease registry 1968–82: Incidence and survival. *Br. J. Cancer*, **56**, 853–858.

CRAFT AW, PARKER L, OPENSHAW S, CHARLTON M, NEWELL J, BIRCH JM AND BLAIR V. (1993). Cancer in young people in the north of England 1968–85: analysis by census wards. *J. Epidemiol. Commun. Health.*, **47**, 109–115.

DOLL R, EVANS HJ AND DARBY SC. (1994). Paternal exposure not to blame. *Nature*, **367**, 678–680.

DRAPER GJ, STILLER CA, CARTWRIGHT RA, CRAFT AW AND VINCENT TJ. (1993). Cancer in Cumbria and in the vicinity of the Sellafield nuclear installation, 1963–90. *Br. Med. J.*, **306**, 89–94.

GARDNER MJ, HALL AJ, DOWNES S AND TERRELL JD. (1987*a*). Follow up study of children born elsewhere but attending schools in Seascale, West Cumbria (schools cohort). *Br. Med. J.*, **295**, 819–822.

GARDNER MJ, HALL AJ, DOWNES S AND TERRELL JD. (1987*b*). Follow up study of children born to mothers resident in Seascale, West Cumbria (birth cohort). *Br. Med. J.*, **295**, 822–827.

GARDNER MJ, HALL AJ, SNEE MP, DOWNES S, POWELL CA AND TERRELL JD. (1990*a*). Methods and basic data of case–control study of leukaemia and lymphoma among young people near Sellafield nuclear plant in West Cumbria. *Br. Med. J.*, **300**, 429–434.

GARDNER MJ, SNEE MP, HALL AJ, POWELL CA, DOWNES S AND TERRELL JD. (1990*b*). Results of case–control study of leukaemia and lymphoma among young people near Sellafield nuclear plant in West Cumbria. *Br. Med. J.*, **300**, 423–429.

GARDNER MJ. (1992). Paternal occupations of children with leukaemia. *Br. Med. J.*, **305**, 715.

HEALTH AND SAFETY EXECUTIVE. (1993). *HSE Investigation of Leukaemia and Other Cancers in the Children of Male Workers at Sellafield*, HSE Books: Sudbury.

HEALTH AND SAFETY EXECUTIVE. (1994). *HSE Investigation of Leukaemia and Other Cancers in the Children of Male Workers at Sellafield: Review of Results published in October 1993*. HSE Books: Sudbury.

INDEPENDENT ADVISORY GROUP. (1984). *Investigation of the Possible Increased Incidence of Cancer in West Cumbria*. HMSO: London.

KENDALL GM, O'HAGAN JA, REES S, WALKER SM AND MUIRHEAD CR. (1988). *Summary of Data held by the National Registry for Radiation Workers*. Report NRPB-R219. HMSO: London.

KINLEN LJ. (1993). Can paternal preconceptional radiation account for the increase of leukaemia and non-Hodgkin's lymphoma in Seascale? *Br. Med. J.*, **306**, 1718–1721.

KINLEN LJ. (1995). Epidemiological evidence for an infective basis in childhood leukaemia. *Br. J. Cancer*, **71**, 1–5.

KINLEN LJ AND JOHN SM. (1994). Wartime evacuation and mortality from childhood leukaemia in England and Wales in 1945–9. *Br. Med. J.*, **309**, 1197–1202.

KINLEN LJ, O'BRIEN F, CLARKE K, BALKWILL A AND MATTHEWS F. (1993). Rural population mixing and childhood leukaemia: effects of the North Sea oil industry in Scotland, including the area near Dounreay nuclear site. *Br. Med. J.*, **306**, 743–748.

KINLEN LJ, DICKSON M AND STILLER CA. (1995). Childhood leukaemia and non-Hodgkin's lymphoma near large rural construction sites, with a comparison with Sellafield nuclear site. *Br. Med. J.*, **310**, 763–768.

LITTLE MP, WAKEFORD R AND CHARLES MW. (1994). A comparison of the risks of leukaemia in the offspring of the Sellafield workforce born in Seascale and those born elsewhere in West Cumbria with the risks in the offspring of the Ontario and Scottish workforces and the Japanese bomb survivors. *J. Radiol. Prot.*, **14**, 187–201.

PARKER L, CRAFT AW, SMITH J, DICKINSON H, WAKEFORD R, BINKS K, McELVENNY D, SCOTT L AND SLOVAK A. (1993). Geographical dstribution of preconceptional radiation doses to fathers employed at the Sellafield nuclear installation, West Cumbria. *Br. Med. J.*, **307**, 966–971.

ROMAN E, WATSON A, BERAL V, BUCKLE S, BULL D, BAKER K AND RYDER H. (1993). Case–control study of leukaemia and non-Hodgkin's lymphoma among children aged 0–4 years living in West Berkshire and North Hampshire health districts. *Br. Med. J.*, **306**, 615–621.

SIMMONDS JR, ROBINSON CA, PHIPPS AW, MUIRHEAD CR AND FRY FA. (1995). *Risks of Leukaemia and Other Cancers in Seascale from all Sources of Ionising Radiation Exposure*. Report NRPB-R276. HMSO: London.

STATHER JW, DIONIAN J, BROWN J, FELL TP AND MUIRHEAD CR. (1988*a*). The risk of leukemia in Seascale from radiation exposure. *Health Phys.*, **55**, 471–481.

STATHER JW, CLARKE RH AND DUNCAN KP. (1988*b*). *The Risk of Childhood Leukaemia near Nuclear Establishments*. Report NRPB-R215. HMSO: London.

STILLER CA, DRAPER GJ, VINCENT TJ AND O'CONNOR CM. (1991). Incidence rates nationally and in administratively defined areas. In *The Geographical Epidemiology of Childhood Leukaemia and Non-Hodgkin Lymphomas in Great Britain, 1966–83*, Draper G. (ed) pp. 25–35. HMSO: London.

UNITED NATIONS SCIENTIFIC COMMITTEE ON THE EFFECTS OF ATOMIC RADIATION. (1994). *Sources and Effects of Ionizing Radiation*. UNSCEAR 1994 Report to the General Assembly, with Scientific Annexes. United Nations: New York.

URQUHART JD, BLACK RJ, MUIRHEAD MJ, SHARP L, MAXWELL M, EDEN OB AND ADAMS JONES D. (1991). Case–control study of leukaemia and non-Hodgkin's lymphoma in children in Caithness near the Dounreay nuclear installation. *Br. Med. J.*, **302,** 687–692.

WAKEFORD R, TAWN EJ, MCELVENNY DM, SCOTT LE, BINKS K, PARKER L, DICKINSON H AND SMITH J. (1994*a*). The descriptive statistics and health implications of occupational radiation doses received by men at the Sellafield nuclear installation before the conception of their children. *J. Radiol. Prot.*, **14,** 3–16.

WAKEFORD R, TAWN EJ, MCELVENNY DM, BINKS K, SCOTT LE AND PARKER L. (1994*b*). The Seascale childhood leukaemia cases–the mutation rates implied by paternal preconceptional radiation doses. *J. Radiol. Prot.*, **14,** 17–24.

WHELDON TE. (1989). The assessment of risk of radiation-induced childhood leukaemia in the vicinity of nuclear installations. *J. R. Stat. Soc. A.*, **152,** 327–339.